# Augmented Reality Playgrounds to Promote Physical Activity in Young Children: Feasibility Study Using a Repeated Measures Laboratory Design

**DOI:** 10.2196/75302

**Published:** 2025-10-09

**Authors:** Sarah M Stearne, Amber Beynon, Charlotte Lund Rasmussen, Juliana Zabatiero, Louise Paatsch, Daniel Johnson, Leon Straker, Amity Campbell

**Affiliations:** 1 Curtin School of Allied Health, Curtin University, Building 401 Kent StreetPerth, 6102, Australia, 61 0892661771; 2 Australian Research Council Centre of Excellence for the Digital Child, Brisbane, Australia; 3 School of Education, Deakin University, Geelong, Australia; 4 School of Computer Science, Queensland University of Technology, Brisbane, Australia

**Keywords:** augmented reality, children, play, physical activity, enjoyment, digital technology, smartphone

## Abstract

**Background:**

Only 10% of Australian children meet the recommended daily physical activity guidelines. Augmented reality (AR) is increasingly being used in primary education and clinical rehabilitation, with high enjoyment and motivation to participate frequently reported. AR has increased physical activity participation in adult populations, but whether AR can increase physical activity in young children has yet to be investigated.

**Objective:**

This study aimed to determine if an indoor AR-enhanced playground is enjoyed by young children and prompts physical activity in both low- and high-structured scavenger hunts.

**Methods:**

Seventeen pairs of 5‐ to 8-year-olds participated in 2 animal search tasks (ie, AR and non-AR) in 2 activity structure levels (ie, low-structured and high-structured) in a 2×2 repeated measures design in an indoor laboratory playground. Children searched for either AR animals (a custom AR app on a smartphone) or toy animals and followed a set obstacle course route (high-structured) or moved wherever they wished (low-structured). Questionnaires assessed child enjoyment, perceived physical activity, and caregiver perception of enjoyment. Thigh-worn accelerometers (SENS; SENS Innovation ApS) assessed postures and movements, and a video camera recorded engagement time.

**Results:**

Children rated AR conditions (low-structured: mean 4.4, SD 1.0, and high-structured: mean 4.5, SD 0.9) as more enjoyable than high-structured non-AR (mean 4.1, SD 1.0; *P*=.03). When asked which condition was the most enjoyable, 15 chose the low-structured AR, followed by the high-structured AR (n=11) and low-structured non-AR (n=8). Caregiver perception of children’s enjoyment ratings generally aligned. Ratings of perceived physical activity level were the same in all conditions (mean 4.3, SD 0.7; *P*>.05). Accelerometry showed that a greater percentage of time was spent in low-intensity postures and movements during AR conditions (AR: mean 50%, SD 13% vs non-AR: mean 35%, SD 14%; *P*<.001), namely in sitting and standing, and in high-intensity movements during non-AR (AR: mean 21%, SD 12% vs non-AR: mean 32%, SD 18%; *P*<.001). During low-structured conditions, engagement time was significantly longer with the AR animals compared to the toy animals (AR: mean 263.1, SD 65.7 seconds vs non-AR: mean 197.3, SD 76.5 seconds; *P*=.002).

**Conclusions:**

While the intensity of physical activity was lower during AR, the greater enjoyment and longer engagement time may lead to greater overall accumulation of active play by motivating young children to go to and stay longer at playgrounds. The high-structured AR conditions resulted in higher-intensity physical activity compared to low-structured AR conditions; however, enjoyment ratings from children and caregivers were generally higher in the low-structured AR. Therefore, AR may be suitable to implement in both low- and high-structured play environments. Future research should investigate whether these findings hold true at outdoor playgrounds and examine the impact of novelty over time.

## Introduction

Augmented reality (AR) digital technology is becoming increasingly available and promoted to children in both home and educational settings [1,2]. AR refers to the use of a smart device with an incorporated camera to present the user with an integrated, real-time view of the real world with superimposed digital information. This is commonly a smartphone or tablet, but headset technology is rapidly advancing, making it more affordable and available [3]. AR applications vary enormously, from static overlaid images to animated 3D creatures to semi-immersive 3D worlds. Not only can the appearance and fidelity of AR vary enormously, but so too can the way it is “activated.” AR can be activated using a physical marker (eg, a QR code or image) or be markerless (eg, using GPS location or world tracking, where the device camera scans the environment for a flat surface to place the AR object upon). Despite its growing popularity, the implications of AR use by young children remain relatively unstudied. While there is often inherent skepticism and concern about potential risks with children engaging with digital technology [4], the potential benefits of AR should also be investigated.

Primary school education, particularly in science, technology, engineering, and mathematics subject areas, can benefit from AR by allowing teachers to extend learning into a 3D space and for children to visualize concepts that would not normally be accessible in real life [5]. Compared with traditional learning resources, children aged 8‐10 years have reported a preference for AR games when learning about multiculturalism, solidarity, tolerance, and the water cycle [6,7]. AR has also been shown to increase motivation to learn [5] and knowledge retention [8] in areas such as mathematics [9], literacy, geography, biology [5], and recycling behavior [10]. The increased appeal and motivation that AR provides for children in educational settings could translate into other aspects of their daily lives, for example, motivating children to be physically active in leisure time.

Children’s participation in physical activity in many countries is generally low. For example, in Australia, parent-reported data show that in 2020, only 10% of children aged 5‐18 years participated in the recommended 60 minutes of moderate-to-vigorous physical activity daily [11]. Sufficient physical activity and avoiding excessive sedentary behavior are vital for child health and well-being by reducing obesity risk [12], improving mental health [13], and supporting muscle and bone development [14]. Enjoyment has been identified as a key factor in children’s participation in physical activity in theoretical models such as the self-determination theory [15]. Intrinsic motivation and higher enjoyment of physical activity are related to increased physical activity levels [16-18]. Further, a randomized controlled trial of 2087 adolescent girls demonstrated that increasing enjoyment of physical activity resulted in increased voluntary participation in physical activity [19].

AR is effective in increasing physical activity levels in adults and adolescents [20,21]. For example, adolescents in a study of Pokémon Go (2016; Niantic Inc), an AR smartphone game that encourages users to move to different geographical locations to “catch” Pokémon, increased their participation in low, moderate, and vigorous physical activity by ~41 minutes per week [22] over 7 weeks. AR has also been shown to increase the motivation of adolescents to participate in physical education classes [23]. AR applications that encourage children to be physically active are becoming more prevalent [24,25]; however, very little research has been undertaken regarding the feasibility and effectiveness of AR to promote physical activity, motivation, and enjoyment among primary school–aged children. However, evidence shows that AR apps can improve motor function and increase participation in physical rehabilitation among pediatric clinical populations [26]. For example, in children with cerebral palsy, AR has been shown to improve upper limb muscle strength and range of motion [27] and to be an effective tool in improving gait speed [28]. In a review [29], the most frequently reported advantage of AR use in a clinical population was increased motivation leading to increased participation. If AR can effectively increase participation in physical rehabilitation in a clinical pediatric population, it remains possible that it could be an effective way to encourage increased physical activity in typically developing primary school–aged children; however, this has not been investigated.

AR as a tool to encourage physical activity in children is attractive due to its easy access and portability, being predominantly used on a smart device (eg, smartphone or tablet) already owned by many families, and its ability for use in both indoor and outdoor environments. Outdoor play not only provides children with the opportunity to practice and refine gross motor skills [30] but also reduces the risk of myopia [31] and vitamin D deficiency, which has been linked to poor bone development, cardiovascular disease, and diabetes [32]. This flexibility of use across multiple environments contrasts with many other digital devices aimed at eliciting active play in children, such as console-based active video games [33]. The versatility of AR applications, particularly marker-based AR, also allows for easy adaptation to different situations, such as different play styles. Physically active play can be broadly grouped into 2 overarching categories: high-structured and low-structured. High-structured physical play requires children to follow a set of rules or activities (eg, an obstacle course or football drills), while low-structured play is child-led free play. While both play types have overall benefits, highly structured play can emphasize motor skill development, while low-structured or free play can enhance imagination, autonomy, confidence, and self-esteem [34]. Marker-based AR could be adapted to both play styles, as the physical markers can, for example, be placed around an obstacle course by a parent, educator, or coach (high-structure play) or around a playground (low-structure play), prompting children to engage with the AR in diverse ways. However, how AR may encourage physical activity in children engaging in different play types remains unknown. This study aimed to explore whether marker-based AR digital technology could be a useful tool for engaging young children in physical activity during both high-structured and low-structured play, compared to a non-AR task. We hypothesized that children would enjoy AR more than non-AR conditions, and that their physical activity level (perceived and measured) would be higher in both high-structured and low-structured play.

## Methods

### Study Design

This was a 2×2 repeated measures study (Animal Format: smartphone AR vs non-AR plastic toys × Activity Structure: low-structure vs high-structure) where children participated in 4 animal scavenger hunts in a laboratory playground setting. Outcome measures included enjoyment, time spent engaging, and level of physical activity. Pilot testing of the study protocol and AR app was conducted with 4 children (2 pairs, aged 5‐8 years, male and female), and the protocol and app were refined before the commencement of participant data collection.

### Laboratory Setup

**Figure 1.**
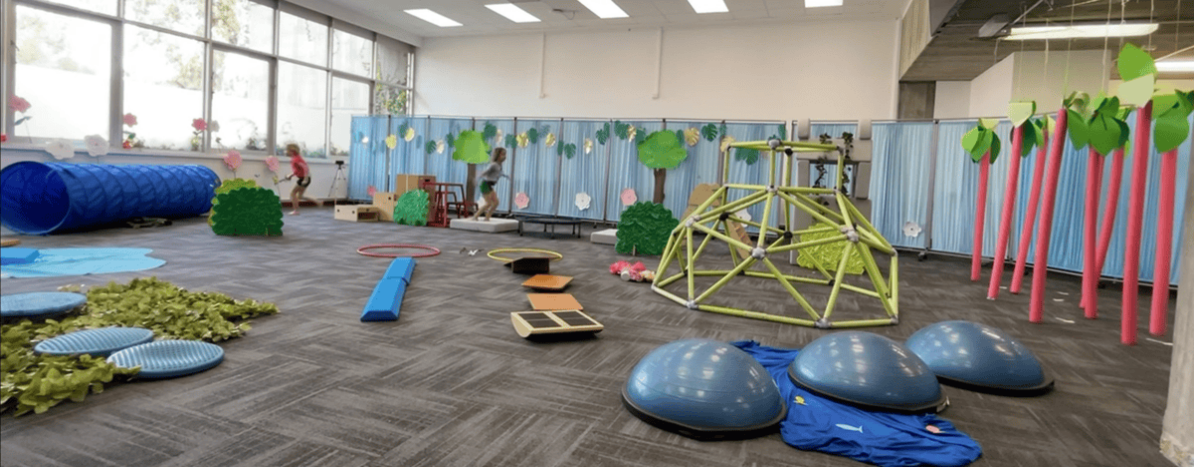
Laboratory play equipment setup (all conditions) showing 2 participants in the low-structure non–augmented reality (non-AR) condition.

The indoor laboratory was set up to simulate a playground, with the following equipment: a soft balance beam, wobble cushions, a see-saw plank, a hula-hoop tunnel, wooden boxes to climb, soft cushions to jump on, a mini trampoline, a climbing frame, a soft tunnel to walk through, hanging pillars to navigate, half Bosu balls, and wobble boards (Figure 1). Equipment setup was standardized for all participants and conditions.

### Participants

Thirty-four children aged 5‐8 years and their caregivers volunteered to participate in the study. They were recruited from the local community and workplace networks in an Australian city via word of mouth and social media advertisements. Eligible children took part in the study in pairs, either a known friend or sibling (both henceforth termed “peer”). Parents and caregivers (henceforth termed “caregiver”) completed a screening questionnaire to determine if they and their children were eligible before being enrolled in the study. Participants were excluded if they did not speak fluent English (child and caregiver), had a physical injury (eg, sprained ankle) that limited their ability to move normally, a history of seizures or epilepsy, binocular vision abnormalities (eg, strabismus or amblyopia), frequent headaches or earaches, experienced severe motion sickness, had a known skin response to medical tape, or had any known or suspected psychological or physical clinical diagnosis that may influence their ability to understand and follow instructions or perform requested activities. The sample size was deemed appropriate based on previous accelerometer studies with children [35].

### Protocol

Children and their caregivers visited the university laboratory for a single data collection session, approximately 60 minutes in duration. A standardized warm-up was completed, which involved a familiarization period with the AR technology, playground equipment, and obstacle course route. Children completed 4 conditions concurrently with their peers. Condition order was randomized (refer to Multimedia Appendix 1). Children were told they could work together with their peers but did not have to; it was their choice.

### Animal Format: AR Versus Non-AR

**Figure 2.**
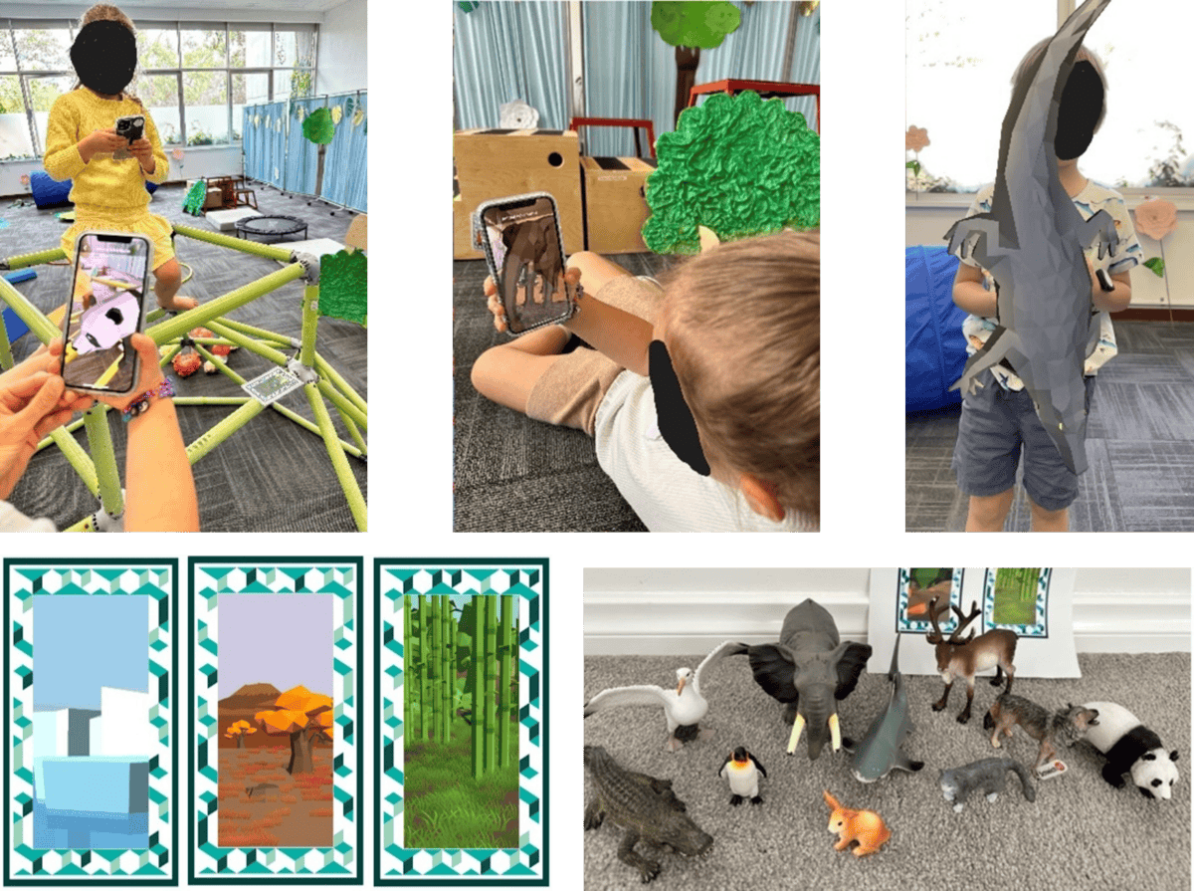
Top shows children playing with the panda, elephant, and crocodile augmented reality (AR) animals during the AR conditions. The bottom left shows the VuMarks (marker-based AR) used in the AR conditions, including the penguin, deer, and panda. The bottom right shows the plastic toy animals used in the non-AR conditions.

A custom marker-based AR application (Animal Adventures; Curtin University) was developed for this study using Unity Vuforia Engine (Unity Technologies). Ten custom VuMarks (similar to a QR code but colorful images of the animal habitats; Figure 2) were designed, each linked to a different AR animal. VuMarks were 12×7.5 cm each. AR animals were virtual 3D low-poly animated animals (Unity Asset Store, Polyperfect) that appeared to be 30-100 cm in height, including a shark, elephant, panda, penguin, cat, rabbit, wolf, crocodile, seagull, and deer (Figure 2). AR animal size was adjusted to ensure they were visually impressive but not too large to be difficult to view on the smartphone. The distance the AR animal appeared relative to the VuMark was adjusted to ensure the children knew the animal had appeared (ie, close enough to the VuMark) but also far enough away to ensure the entire animal was visible. Once VuMarks were activated by holding them in the phone’s camera view for one second, the AR animal remained visible in that location for the remainder of the condition. The decision to use this setting was to prevent the animal from disappearing while children were viewing and interacting with it, which otherwise resulted in frustration. It also created the illusion of being surrounded by animals at the playground. Children were provided with an Apple iPhone 12 (Apple Inc) each to use during the AR conditions. Children were given a demonstration and the opportunity to try using the AR app before commencing the first AR condition. For the non-AR conditions, plastic toy animals (Schleich Inc), the same as the AR animals, which ranged in size from 3‐20 cm, were used (Figure 2).

### Scavenger Hunt Activity Structure: Low-Structure Versus High-Structure

Conditions involved searching for 6 animals around the indoor play equipment, either AR animals or toy animals (non-AR), depending on the condition. Children were instructed that they could move wherever they wanted to find the animals during the low-structure conditions, or follow a set obstacle course route during the high-structure conditions. During the high-structure obstacle course conditions, children completed 2 laps of the circuit at the same time as their peers but in the opposite direction. They were not given any specific instructions on time, only that they should try to find the 6 animals while completing the 2 laps. During low-structure conditions, children were told there were 6 animals to find, after which they could play until they were told time was up. The low-structure conditions ended after a maximum of 5 minutes or earlier if the children lost interest in playing (total time). The time children stopped engaging with the AR or the non-AR animals during the low-structure conditions was noted and referred to as “engagement time.”

### Data Collection Tools

#### Questionnaires

Children completed a series of questionnaires for the outcomes of enjoyment and perceived physical activity. Children and caregivers were blinded to the hypotheses of the research in an attempt to avoid biases such as social desirability bias. Caregivers completed questionnaires regarding the outcomes of perceived enjoyment and their child’s previous digital technology experience.

Child enjoyment and caregiver perception of their child’s enjoyment were recorded following each condition and after the conclusion of all conditions using items from the Fun Toolkit [36], which have been frequently used with young children [37]. Specifically, children and caregivers were invited to circle the best response on a 5-item Smileyometer [37], a pictorial scale with round yellow faces ranging from awful (=1) to brilliant (=5) (refer to Multimedia Appendix 1) at the end of each condition. In addition, children and caregivers were invited to complete 3 variations of the fun sorter [37] following all 4 conditions. First, they were asked to rank which animal search condition they enjoyed more by placing cut-out images representing the conditions (eg, AR or non-AR animals) into the appropriate box. Second, they were invited to rate whether they found the low-structure or high-structure conditions more enjoyable. Finally, they were asked to rank all 4 conditions from most enjoyable to least enjoyable. Caregivers were also provided with the option to complete a written open-ended question after each fun sorter task to elaborate on the reason for their answers.

Following each condition, children completed a 5-item pictorial Perceived Physical Activity Rating Scale, selecting how physically active they felt they were during the condition, from completely stationary (=1) to fast running (=5). This scale was adapted from the Children’s Physical Activity Rating Scale (CARS) [38], a validated measure of observer-rated physical activity levels in young children. The adapted scale used the same 5 levels as the CARS but included illustrations, and children completed the scale themselves (refer to Multimedia Appendix 1). Caregivers were asked to complete a short questionnaire about their child’s previous experience with digital technology.

#### Video

Total time and engagement time were calculated from accelerometer data and video footage. Video footage was captured from 2 video cameras (model A2403; Apple Inc; 30 Hz super wide lens) affixed to 1-meter-tall tripods and placed at opposite edges of the play area. Low structure condition total time (AR and non-AR conditions) commenced when the children started the condition and ended when either they lost interest in playing or after 5 minutes, whichever was sooner. Low structure condition engagement time (AR and non-AR) commenced when the child started the condition and ended when they lost interest in playing with the AR app or toys. In high-structure conditions, total time and engagement time were the same, commencing when the children started the obstacle course and ending when they completed their 2 circuits.

#### Accelerometer

The time children spent in different postures and movements was estimated from a thigh-worn accelerometer (SENS Innovation ApS) previously used in research with children of the same age [35]. The SENS motion PLUS (model number A01.6) accelerometer weighs 7 g and is 47×22×4.5 mm in size and was placed on the midline of the child’s anterior right thigh halfway between the hip and knee. It was affixed using hypoallergenic double-sided tape underneath the accelerometer and hypoallergenic Fixomull stretch over the top. It was worn for the entire data collection. The accelerometer operated at 12.5 Hz and recorded accelerations in the x, y, and z axes with a range of +4 to −4 G.

### Analysis

#### Accelerometer Analysis

Accelerometer data were cropped using a customized LabVIEW program (National Instruments). The Motus software, based on the Acti4 algorithm as developed and described in Skotte et al [39] and validated for use in school-aged children in a laboratory [35,40], was used to classify postures and movements. The software resamples the accelerometer data to 30 Hz in 2-second windows with a 50% overlap and uses both the inclinations and SDs of the thigh accelerations to classify different postures and movements using a rule-based algorithm [39]. The classified postures and movements are lying, sitting, standing, moving (stationary but with fidgeting legs), walking, running, stair climbing, cycling, or rowing (high-intensity movement that is not classified as running, stairs, or cycling). A customized R program (R Development Core Team) summarized these data into total and percentage of time spent in each movement and posture per condition. Due to the minimal incidences of movement coded as cycling and rowing (average total across all 4 conditions was 4.9, SD 10.1 seconds, and mean 4.6, SD 15.4 seconds, respectively, of a total average of 1200.4, SD 130.2 seconds), they were combined with the stair-climbing time to create a single stairs+ variable. Postures and movements were grouped as low intensity (lying, sitting, standing, and moving), moderate intensity (walking), and high intensity (running and stairs+).

#### Statistical Analysis

Questionnaire data from children and caregivers, along with pictorial scale numerical values, were transcribed into Microsoft Excel (Microsoft Corporation), and descriptive statistics were calculated. All other statistical analyses were performed in Jamovi (version 2.3.28; Jamovi). A critical alpha probability level was set at *P*<.05 for all analyses. Two-by-two (2×2) repeated-measures ANOVAs with post hoc pairwise comparisons without Bonferroni correction were run for all measures, except binomial fun sorter data, where a chi-square analysis was used. A thematic analysis of caregiver open-ended questionnaire responses was conducted.

### Ethical Considerations

The study was approved by the Curtin University Human Research Ethics Committee (HRE2023-0323) before its commencement. An information sheet was provided to caregivers, and time was allowed for questions before the caregiver provided written consent. A simplified version of the information sheet was shown to children with their caregiver present. The study procedures were explained to children using visual illustrations and demonstrations of the study equipment, and time was allowed for questions. Children were invited to provide their verbal and written assent to participate. Three researchers were present for data collection, and all had current Working with Children Clearances. All data were deidentified for analysis when possible.

## Results

### Participants

All 34 children completed the study. Their average age was 6.2 (SD 1.2) years (range 5‐8 years), 41% (n=14) were female, and all were currently in primary school education. The majority (n=20, 59%) attended with a friend of the same age and gender, while 41% (n=14) attended with a sibling (4 male-female sibling pairs, 2 brother pairs, and 1 sister pair). Parents reported that 47% (n=16) of children had previous experience with AR, most notably Pokémon Go, 59% (n=20) had active video game experience, and 94% (n=32) had previously played sedentary games on a computer, tablet, or smartphone. There was no correlation between age and AR experience (*r*_32_=0.165; *P*=.35).

### Enjoyment in AR Versus Non-AR

**Figure 3.**
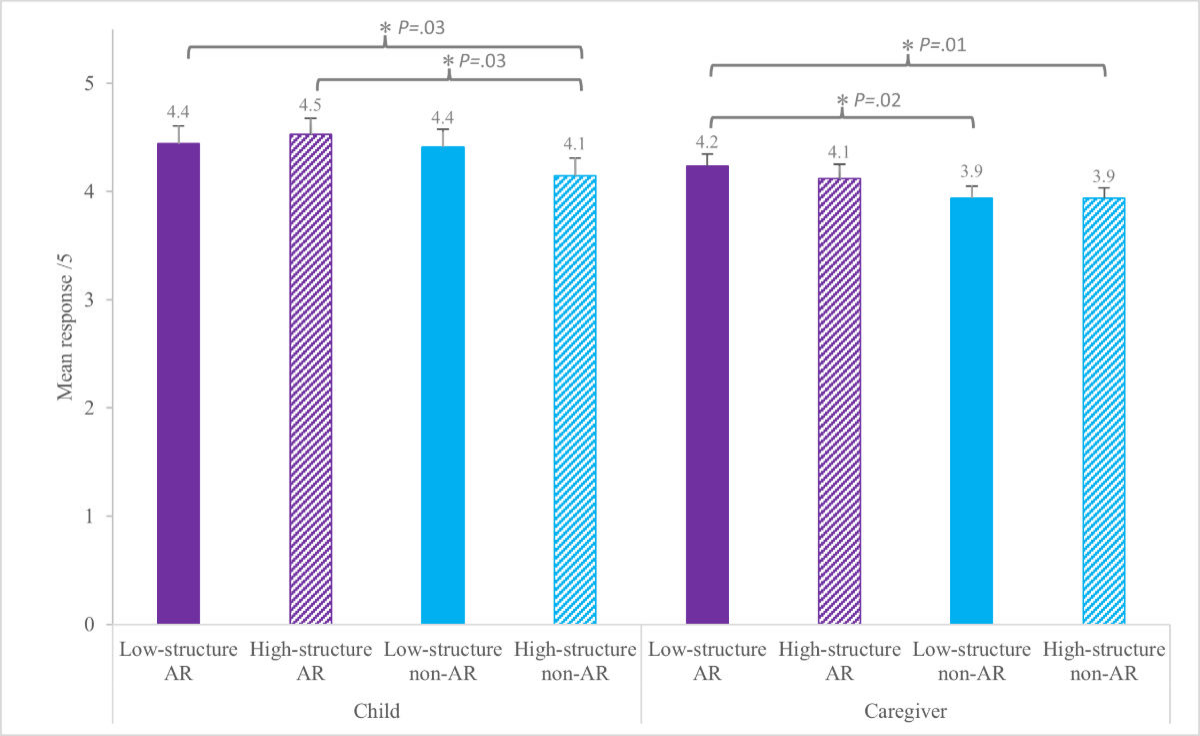
Group mean (SE) for child enjoyment and caregivers’ perception of their child’s enjoyment on the Smileyometer scale of 1 to 5, where 1 was “awful” and 5 was “brilliant.” AR: augmented reality. *Indicates statistically significant differences in pairwise comparison (*P*<.05).

Results from the children’s ratings on the 5-item Smileyometer showed that AR was generally rated as more enjoyable than non-AR, and low-structure was sometimes rated as more enjoyable than high-structure. For child ratings of enjoyment, there was no overall main effect of Animal Format (AR: mean 4.5, SD 0.9 vs non-AR: mean 4.3, SD 1.0; *F*_1,33_=3.79; *P*=.06) or Activity Structure (low-structure: mean 4.4, SD 0.9 vs high-structure: mean 4.3, SD 0.9; *F*_1,33_=0.90; *P*=.35). However, pairwise comparisons revealed children rated low-structure AR and high-structure AR as more enjoyable than high-structure non-AR (*t*_33_=2.26; *P*=.03; *d*=0.03 and *t*_33_=2.26; *P*=.03; *d*=0.32, respectively; Table 1 and Figure 3). Caregivers’ perception of their child’s enjoyment was similar, though with a significant main effect of Animal Format showing AR rated as more enjoyable (AR: mean 4.2, SD 0.7 vs non-AR: mean 3.9, SD 0.6; *F*_1,33_=6.81; *P*=.01; *d*=0.46), but no main effect of Activity Structure (low-structure: mean 4.1, SD 0.7 vs high-structure: mean 4.0, SD 0.7; *F*_1,33_=0.66; *P*=.42). Pairwise analysis showed that caregivers rated low-structure AR as more enjoyable than low-structure non-AR and high-structure non-AR (*t*_33_=2.54; *P*=.02; *d*=0.50 and *t*_33_=2.73; *P*=.01; *d*=0.54; respectively; Table 1 and Figure 3). Multimedia Appendix 1 displays results based on the number of responses.

**Table 1.** Enjoyment, time, and physical activity. Superscript letters indicate statistical groupings. Values within a row that share a letter are not statistically different from each other. Conversely, values with no letters in common are significantly different (*P*<.05)

	Augmented reality (AR), mean (SD)		Non-AR, mean (SD)	
Outcome	Low-structure	High-structure	Low-structure	High-structure
Child enjoyment (scale 1-5, higher number=greater enjoyment)	4.4 (1.0)^a^	4.5 (0.9)^a^	4.4 (1.0)^a, b^	4.1 (1.0)^b^
Caregiver perception of enjoyment (scale 1-5, higher number=greater enjoyment)	4.2 (0.6)^a^	4.1 (0.8)^ab^	3.9 (0.6)^b^	3.9 (0.5)^b^
Child fun sorter (1-4 rank, higher number=greater enjoyment)	3.2 (0.9)^a^	2.8 (1.1)^a, b^	2.4 (1.1)^b^	1.6 (0.8)^c^
Caregiver fun sorter (1-4 rank, higher number=greater enjoyment)	3.4 (0.9)^a^	2.7 (0.8)^b^	2.2 (1.1)^b^	1.7 (0.9)^c^
Total time (seconds)	329.6 (22.0)^a^	225.7 (69.4)^b^	312.5 (61.1)^a^	158.1 (61.2)^c^
Engagement time (seconds)	263.1 (65.7)^a^	225.7 (69.4)^b^	197.3 (76.5)^b^	158.1 (61.2)^c^
Child physical activity perception (scale 1-5, higher number=greater percived activity)	4.3 (0.7)	4.3 (0.7)	4.3 (0.8)	4.4 (0.8)
Total percentage low-intensity exercise (combination of lie, sit, stand, and move)	54 (11)^a^	47 (13)^b^	37 (12)^c^	33 (15)^d^
Total percentage moderate-intensity exercise (walk)	28 (9)^a^	30 (11)^a^	34 (12)^b^	30 (13)^a, b^
Total percentage high-intensity exercise (run and stairs+)	18 (11)^a^	24 (12)^b^	29 (15)^c^	37 (20)^d^
Lie (%)	2 (5)^a^	1 (2)^b^	3 (5)^a^	1 (4)^a, b^
Sit (%)	11 (8)^a^	8 (9)^a^	4 (4)^b^	3 (4)^b^
Stand (%)	12 (8)^a^	7 (6)^b^	4 (4)^c^	3 (3)^c^
Move (%)	29 (6)^a, b^	31 (9)^b^	27 (9)^a^	26 (0.1)^a^
Walk (%)	28 (9)^a^	30 (11)^a^	34 (12)^b^	30 (13)^a, b^
Run (%)	12 (10)^a^	13 (12)^a^	23 (14)^b^	28 (19)^c^
Stairs+ (%)	7 (6)^a^	11 (7)^b^	6 (5)^a^	8 (7)^a, b^

a Groups with no statistical significance in pairwise comparison (*P*>.05).

b Groups with no statistical significance in pairwise comparison (*P*>.05).

c Groups with no statistical significance in pairwise comparison (*P*>.05).

d Groups with no statistical significance in pairwise comparison (*P*>.05).

In the fun sorter questionnaire comparing enjoyment in AR and non-AR conditions (or caregivers’ perception of their child’s enjoyment), both children and caregivers ranked AR higher (children: 82%, n=28, *χ*^*2*^_1,34_=15.1; *P*<.001 and caregivers: 91%, n=31, *χ*^2^_1,34_=21.1; *P*<.001). When comparing enjoyment during low and high-structured conditions, both children and caregivers ranked low-structured conditions higher (children: 69%, *χ*^2^_1,34_=4.5; *P*=.03 and caregivers: 69%, *χ*^2^_1,34_=4.5; *P*=.03). There was a significant negative correlation between previous AR experience and AR enjoyment (*r*_32_=−0.366; *P*=.03), with those who had not experienced AR previously more likely to rate it as the more enjoyable activity over the non-AR. Younger children (5 or 6 years old) were also more likely to rate the AR experience as more enjoyable (*r*_32_=−0.366; *P*=.03) than older children (7 or 8 years old).

**Figure 4.**
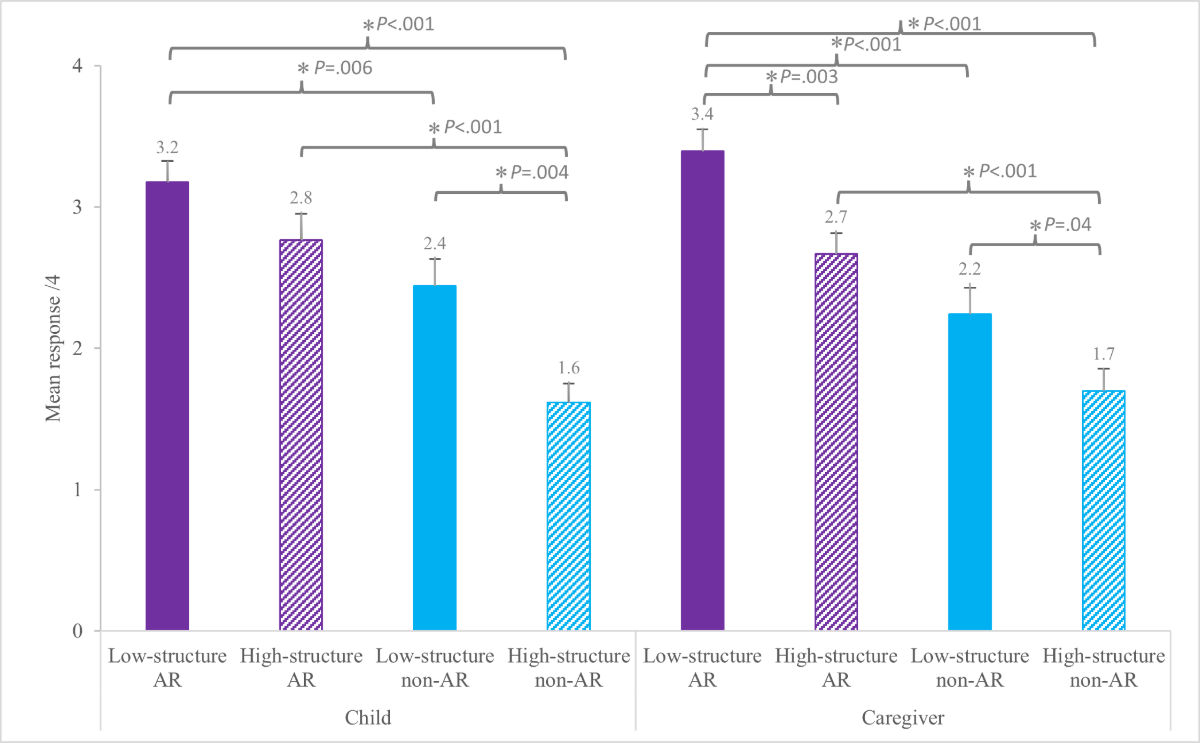
Group mean (SE) for child enjoyment and caregivers’ perception of their child’s enjoyment on the fun sorter ranking scale of all 4 conditions, where 4 was the favorite condition and 1 was the least favorite. AR: augmented reality. *Indicates statistically significant differences in pairwise comparison (*P*<.05).

When children and caregivers were asked to rank the enjoyment of all 4 conditions individually, there were main effects of Animal Format and Activity Structure for both children (*F*_1,33_=22.65; *P*<.001 and *F*_1,33_=7.13; *P*=.01, respectively) and caregivers (*F*_1,33_=33.21; *P*<.001 and *F*_1,33_=7.61; *P*=.01, respectively). The majority of children and caregivers ranked low-structure AR as the most enjoyable condition (n=15 and n=21, respectively), followed by high-structure AR (n=11 and n=6, respectively; Table 1, Figure 4, and Multimedia Appendix 1).

Of the 31 caregivers who rated AR as preferred, 25 chose to provide a written rationale for their rankings. Forty-eight percent (12/25) of caregivers wrote their child demonstrated more signs of excitement during the AR conditions, for example, stating, “More expressive, finding the AR animals ‘argh, the shark is scary.’” Forty-eight percent (12/25) of caregivers who rated AR as preferred also referred to the novelty of the AR experience, making it more enjoyable, for example, stating, “[child] has never used AR, I think the novelty of it was a bit more enjoyable for him (new always better than old).” Caregivers also wrote that being able to use technology made the AR more appealing to their children (8/25, 32%), for example, “[child] hasn’t used smartphone apps and it’s extra exciting to have the technology and AR for the first time.” Two caregivers mentioned that AR enhanced the social connection between their children, stating, “I think it added another dynamic to the play and added a social element.” Of the 3 caregivers who felt their child preferred the non-AR conditions, all 3 believed this was because they were easier to find, stating, “quicker reward, easier to find.” Regarding activity structure, 19 caregivers provided rationales for their responses. Of these, 42% (n=8) believed their child enjoyed the low-structure conditions more because they allowed more freedom and autonomy in play, for example, stating, “a bit more autonomy. They can create their own paths and adventures.” Twenty-one percent (n=4) commented on the increased social aspects of low-structure, for example, stating, “We were able to concentrate on finding animals rather than completing the obstacle items and able to interact more with each other rather than being on their separate courses.”

### Time Spent in Each Condition (Engagement Time)

**Figure 5.**
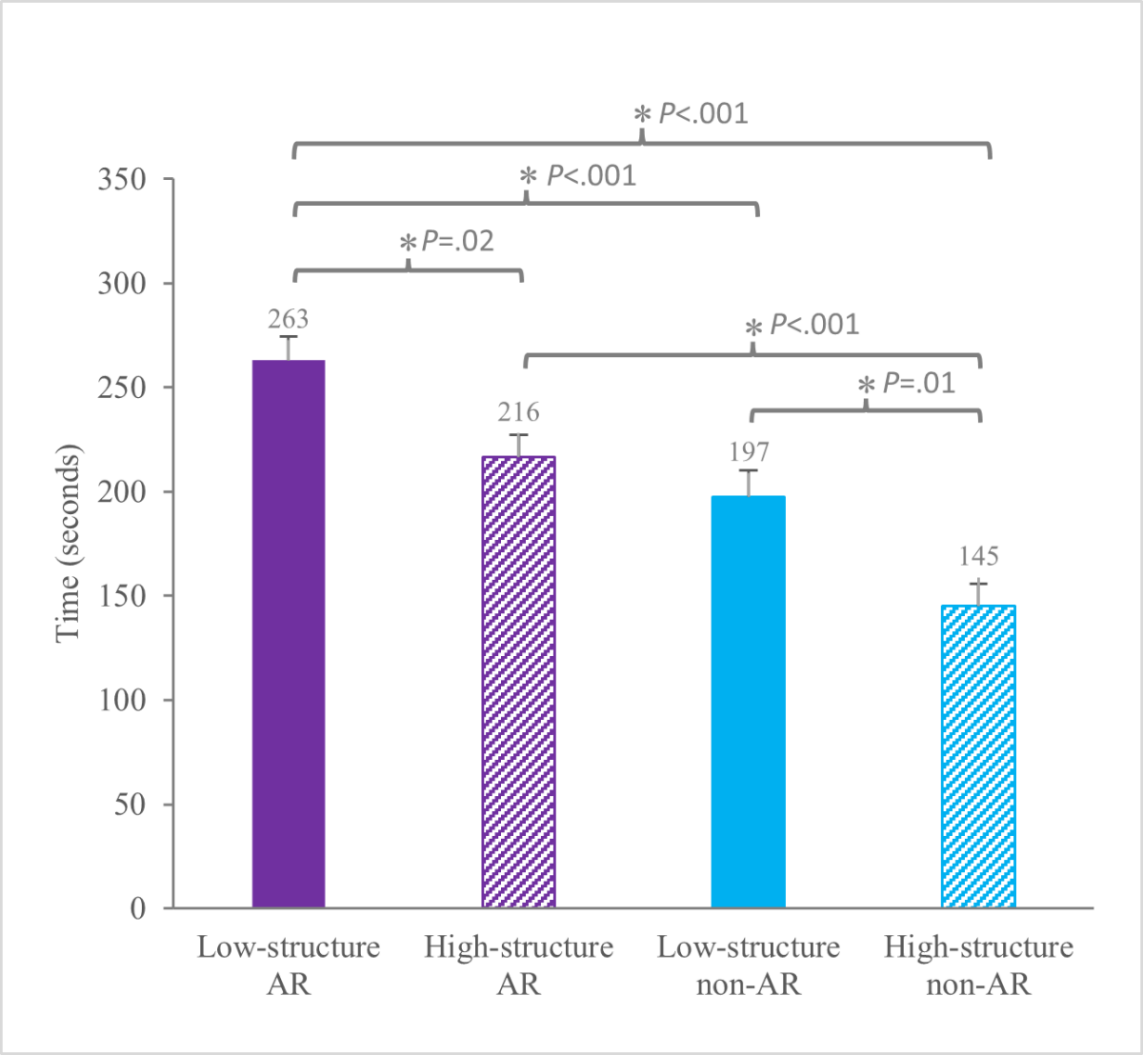
Group mean (SE) for engagement time in seconds. AR: augmented reality. *Indicates statistically significant differences in pairwise comparison (*P*<.05).

Time spent engaging with AR or non-AR animals differed significantly with main effects of both Animal Format (AR: mean 239.8, SD 68.3 seconds vs non-AR: mean 171.3, SD 73.9 seconds; *F*_1,33_=39.17; *P*<.001) and Activity Structure (low-structure: mean 230.2, SD 78.2 seconds vs high-structure: mean 180.9, SD 71.8 seconds; *F*_1,33_=14.14; *P*<.001). Low-structured AR engaged children for longer than all other conditions (mean 263.1, SD 65.7 seconds), followed by high-structured AR (mean 225.7, SD 69.4 seconds; Table 1 and Figure 5).

### Physical Activity Intensity Across Conditions

#### Questionnaire: Perception

Child ratings of physical activity perception showed no main effect of Animal Format (AR: mean 4.3, SD 0.7 vs non-AR: mean 4.3, SD 0.8; *F*_1,33_=0.03; *P*=.86) or Activity Structure (low-structure: mean 4.3, SD 0.7 vs high-structure: mean 4.3, SD 0.8; *F*_1,33_=0.30; *P*=.59; Table 1) on perceived physical activity levels. Pairwise comparisons found no significant results. The majority of participants rated their perceived physical activity level as 5 out of 5 in all conditions, indicating they felt they spent the majority of their time running (refer to Multimedia Appendix 1 and Figure 3).

#### Accelerometer: Percentage of Time Spent in Postures and Movements

##### Low-Intensity Percentage

**Figure 6.**
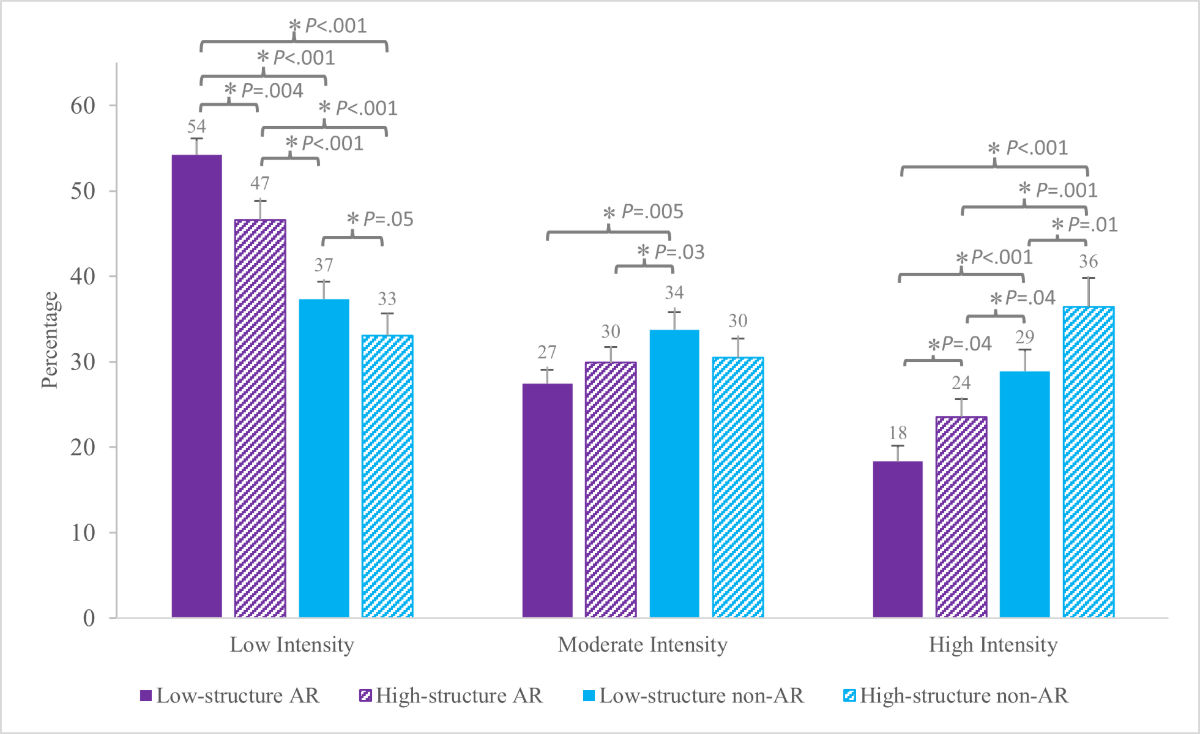
Group mean (SE) accelerometer results for the percentage of time spent in low-intensity postures and movements (lie, sit, stand, and move), moderate intensity (walking), and high intensity (run and stairs+) per condition. AR: augmented reality. *Indicates statistically significant differences in pairwise comparison (*P*<.05).

**Figure 7.**
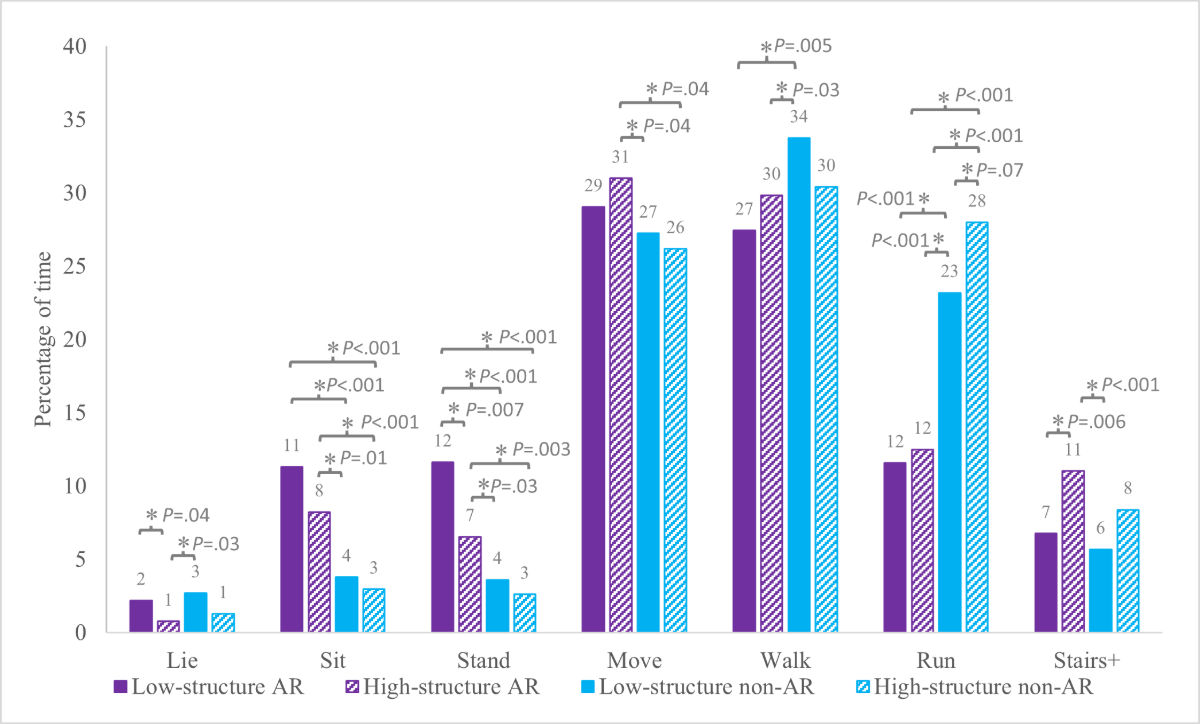
Group mean (SE) accelerometer results for the percentage of total time spent in each posture and movement per condition. AR: augmented reality. *Indicates statistically significant differences in pairwise comparison (*P*<.05).

There were main effects of Animal Format (AR: mean 50%, SD 13% vs non-AR: mean 35%, SD 14%; *F*_1,33_=68.13; *P*<.001) and Activity Structure (low-structure: mean 46%, SD 14% vs high-structure: mean 40%, SD 16%; *F*_1,33_=13.15; *P*<.001) on the percentage of time spent in low-intensity postures and movements. During low-structure AR, the majority of time (54%) was spent in low-intensity postures and movements, a greater percentage than all other conditions (vs high-structure AR, *P*=.004; vs low-structure non-AR, *P*<.001; vs high-structure non-AR, *P*<.001). High-structure AR (47%) also differed from all other conditions (vs low-structure AR, *P*=.004; vs low-structure non-AR, *P*<.001; vs high-structure non-AR, *P*<.001; Figure 6). This was driven primarily by increased time spent standing (12% and 7%, respectively; ) and sitting (11% and 8%, respectively; Table 1 and Figure 7). The lowest percentage of time spent in low-intensity postures and movements was during high-structure non-AR (33%).

##### Moderate-Intensity Percentage

There was a main effect of Animal Format (AR: mean 29%, SD 10% vs non-AR: mean 32%, SD 13%; *F*_1,33_=6.61; *P*=.01) on the percentage of time spent in moderate-intensity movements, but no main effect of Activity Structure (low-structure: mean 31%, SD 11% vs high-structure: mean 30%, SD 12%; *F*_1, 33_=0.12; *P*=.73). A greater proportion of time (34%) was spent walking during low-structure non-AR compared to both AR conditions (low-structure AR: 27%; *t*_33_=3.02; *P*=.005; *d*=0.60 and high-structure AR: 30%; *t*_33_=2.32; *P*=.03; *d*=0.36) but did not differ from high-structure non-AR (30%; *t*_33_=1.69; *P*=.10; Table 1; Figures6  7).

##### High-Intensity Percentage

There were main effects of Animal Format (AR: mean 21%, SD 12% vs non-AR: mean 32%, SD 18%; *F*_1,33_=37.87; *P*<.001) and Activity Structure (low-structure: mean 24%, SD 14% vs high-structure: mean 30%, SD 18%; *F*_1,33_=9.84; *P*=.004) on the percentage of time spent in high-intensity movements. The greatest proportion of time in high-intensity movements was during non-AR conditions, with 36% of time during high-structure non-AR and 29% in low-structure non-AR ( Figure 6). Both were due to an increased percentage of time spent in running (28% and 23%, respectively; Table 1 and Figure 7). The lowest proportion of time spent in high-intensity movements was during low-structure AR (18%; vs high-structure AR: 24%, *P*=.04; low-structure non-AR: 29%, *P*<.001; high-structure non-AR: 37%, *P*<.001). Accelerometer results in seconds are presented in Multimedia Appendix 1.

## Discussion

### Principal Findings

This study was the first to investigate the effect of a marker-based AR-enhanced versus traditional non-AR laboratory-based playground scavenger hunt on young children’s enjoyment and physical activity during both low-structured and high-structured play. Our hypothesis that children would enjoy the AR was supported, with children rating AR in both low- and high-structured scavenger hunts as more enjoyable than non-AR, and they spent longer engaging with the AR. Caregivers’ perceptions of their child’s enjoyment showed a similar pattern. Physical activity was of a lower intensity during AR conditions; however, given the nature of AR interaction requiring children to be stationary to “activate” it, this is not surprising. Despite the lower intensity of activity, the longer engagement time during AR conditions may lead to greater accumulation of active play and, therefore, health and wellness benefits, although this warrants further investigation.

Enjoyment is reported to be a key factor in children’s motivation to participate in physical activity from both theoretical models, such as self-determination theory [15], and research evidence [14,16,18,19,41,42]. In this study, children’s enjoyment ratings across all tasks were high (range 4.1‐4.5 out of 5), but when they were invited to rank conditions, there was a strong preference for AR, with 82% (n=28) of children and 91% (n=31) of caregivers ranking searching for AR animals as more enjoyable than searching for non-AR animals. Condition order was randomized, suggesting the preference for AR was unlikely to be a recency or order effect; however, it is possible there was a novelty effect. Children with no previous AR experience (59%, n=20) were more likely to rate the AR as more enjoyable. In addition, caregivers who ranked AR as more enjoyable commented on the novelty of the AR (48%, n=12) as a possible explanation. This is consistent with research in adults; for example, one study found that most adult participants increased physical activity for only the first 3 to 4 weeks of playing Pokémon Go [43]. How long the potential novelty effect of AR lasts in children is unknown, but could potentially be overcome by the design of the AR application (eg, changing challenges with increased play). Younger children in our study (aged 5-6 years) were more likely to rate the AR as more enjoyable than the non-AR condition in comparison to older children (aged 7-8 years). Given the lack of correlation between age and AR experience, this may be explained by the design of the AR—solo, low-fidelity, slightly animated animals—being too simple to hold the interest of some older children. The AR application used for this study was custom-built for this project by a single programmer and may not be as sophisticated as commercially available products. AR fidelity is an important consideration for future studies, particularly those aimed at an older age group. Caregivers (32%, n=8) also commented on the appeal of technology use as a possible explanation for their child’s high enjoyment. The link between technology use, task enjoyment, and motivation to participate has been noted in children in clinical [29] and educational [5] settings and aligns with the self-determination theory and research by Sebire et al [18] that found young children’s enjoyment was strongly linked with increased participation in physical activity.

While the enjoyment results suggest that AR may be a useful tool to motivate children to participate in physically active play, the physical activity intensity recorded in this study during AR was low. A greater percentage of time was spent in low-intensity postures and movements during AR, in particular low-structured AR, which saw 11% and 12% of time spent in sitting and standing, respectively, compared to 3%‐4% during non-AR conditions. In contrast, a greater percentage of time was spent in high-intensity movements during non-AR conditions, with 23% and 28% of time spent running in low- and high-structured non-AR conditions, respectively, compared to 12%‐13% during AR conditions. Despite the discrepancies in low- and high-intensity postures and movements, the greatest percentage of time in all conditions was spent moving (any instance when the child was stationary but not still, eg, fidgeting) or walking. Given the nature of the AR task, requiring children to stop and remain stationary to “activate” the AR and subsequently engage with it (eg, lying down to pretend the animal was standing on them), the accelerometer physical activity results are not surprising. It should also be noted that during AR conditions, children did participate in high-intensity movements, with a similar percentage of time spent running as they spent standing. While this particular AR experience may not have prompted as much high-intensity physical activity as non-AR play, it did engage children for 36% longer; if this were extrapolated out to a 1-hour playground visit, this could lead to an additional 22 minutes of play. However, future field-based studies with unrestricted time limits are needed to explore this further. Interestingly, children perceived their physical activity level to be comparable across all conditions and felt they spent the majority of their time running. This discrepancy in reported versus recorded physical activity levels, possibly explained by social desirability bias, is common in research [44] and highlights the need for objective measures such as accelerometers to be used in children’s physical activity research [35,40].

AR may be beneficial to implement in both low- and high-structured play environments. The level of activity structure affected physical activity levels, with a greater percentage of time spent in low-intensity postures and movements in low-structure AR conditions, and a greater percentage of time spent in high-intensity movements during the high-structure AR. This finding, however, may be offset by the generally greater enjoyment reported during the low-structured play, potentially leading to greater motivation to participate for longer. AR is versatile and can be adapted to different environments in comparison to many other digital technologies that encourage active play in children, such as game consoles. While these digital devices have been found to elicit low to moderate physical activity levels during gameplay [45], they are restricted to indoor use and require the purchase of expensive equipment. Marker-based AR, such as that used in this study (as opposed to markerless and GPS-based AR), may be particularly useful as it allows the greatest user control over AR location. For example, this study incorporated marker-based AR into laboratory play equipment, but this could easily be relocated to any other indoor or outdoor playground or obstacle course. AR applications may present a cost-effective way to enhance existing community playground infrastructure. Its simple and versatile application could be implemented by coaches and parents to encourage children to participate in certain movements; for example, AR markers could be placed along a beam to encourage balance, at the top of stairs to encourage climbing, or in a low-structured environment to encourage imagination. However, future research should investigate the potential risk to children colliding with objects and tripping over while moving around play equipment with a smartphone. Smartphone AR use by adults has been associated with poorer road-crossing behaviors [46], but whether the risk of miscoordination exists when children use a smartphone for AR remains unknown and warrants investigation before AR playgrounds can be considered in the community.

### Limitations and Future Research

This study had several limitations. The use of subjective ratings in young children may be biased. For example, there were instances where the responses given by some of the children did not match what the researcher or caregiver perceived based on the child’s behavior. These instances were not removed from the dataset and were instead considered a limitation of collecting subjective ratings in young children. Another potential limitation of subjective self-report measures completed in the presence of the researcher is the potential for social desirability bias. This may have impacted the perceived physical activity level and enjoyment outcomes; however, children were blinded to the hypotheses of the research in an attempt to reduce this. Future research should consider looking at evidence of children’s enjoyment of AR playgrounds beyond self-rating scales and caregiver perspectives and using objective measures of physical activity such as accelerometers. This study was limited to a relatively small population in one geographical region and was conducted under controlled laboratory conditions. Whether these findings remain consistent in a community outdoor playground, where children may behave differently, and in different geographical regions, warrants investigation. It should also be acknowledged that the methods used to classify postures and movements from the accelerometer required the child to be performing the movement (ie, running) for at least one full second. Given the small indoor space and distances between play equipment, high-intensity movements may have been underestimated in all conditions. In an outdoor environment with more space to move, physical activity may be of higher intensity. AR experiences vary enormously, from a static generated image to completely immersive environments where the real-world visibility is minimal. Future research should investigate how children of different ages respond to different levels of AR immersion and whether there are added benefits to increased immersion, such as additional enjoyment or increased physical activity, or if it poses risks such as simulator sickness or falling. Simulator sickness is a common side effect in immersive virtual reality and headset AR [47], but little is known about whether handheld AR poses the same risk to children. An additional risk that should be investigated before smartphone AR games are used in community playgrounds is the distraction and social isolation that the presence of a smartphone may introduce. The potential effect that novelty may have on enjoyment, engagement time, and physical activity results should also be acknowledged. As this study was conducted in a single data collection session with a novel AR app, future longitudinal studies are needed to investigate how the novelty of AR-enhanced playgrounds changes over time. Future field-based studies are also needed to determine if unrestricted play time results in greater accumulated physical activity over time when using AR. Practical considerations for playground and AR designers that arose during this study include fidelity of the AR experience to appeal to a wide age range, the added flexibility and control of marker-based AR, and the potential need to maintain interest after initial novelty has worn off.

### Conclusion

This study found that young children enjoyed participating in a search task for AR animals using a smartphone more than searching for traditional toy animals. Given that enjoyment is a key element in children’s motivation to participate in tasks [5], including physical activity [41], AR may have the potential to encourage children to be physically active. While physical activity was of a lower intensity during the AR condition, which may be explained by the nature of the AR requiring children to stand to activate it, the engagement time was longer, potentially leading to an overall greater accumulation of active play if time was not restricted. This study cannot rule out the possibility that the novelty of AR may have produced the high enjoyment reported; therefore, future studies should explore the longer-term effects of AR on enjoyment and physical activity and whether these findings translate from the laboratory to a community outdoor playground. However, these laboratory-based findings suggest that smartphone AR technology could be a novel method to encourage young children to participate in active play.

## Supplementary material

10.2196/75302 Multimedia Appendix 1 Additional material with reviewers’ comments incorporated without track changes.

## Abbreviations

AR: augmented reality
CARS: Children’s Physical Activity Rating Scale
