# Supplementary material for: Augmented Reality Playgrounds to Promote Physical Activity in Young Children: Feasibility Study Using a Repeated Measures Laboratory Design
Source: JMIR Pediatr Parent. 2025 Oct 9;8:e75302. doi: 10.2196/75302 (PMC12510435; doi:10.2196/75302)
Supplement: Multimedia Appendix 1 [file pediatrics-v8-e75302-s001.docx]

**Supplementary material**

**Title:** Getting young children moving in augmented reality playgrounds: a feasibility repeated measures laboratory study.

**Authors:** Sarah Stearne^1,2^, Amber Beynon^1,2^, Charlotte Lund Rasmussen^1,2^, Juliana Zabatiero^1,2^, Louise Paatsch^2,3^, Daniel Johnson^2,4^, Leon Straker^1,2^ and Amity Campbell^1,2^

**Questionnaire’s**

Enjoyment questionnaire (1.) was completed by children and caregivers after each condition and the perceived physical activity (2.) was completed by children following each condition.

1. Enjoyment

How much did you **enjoy** that activity?

2. Physical Activity

How much did you feel you were moving around during that activity?

**Condition Order**

**Supplementary Table 1.** Number of times each condition was performed 1^st^, 2^nd^, 3^rd^, or 4^th^

|  |  | **1st** | **2nd** | **3rd** | **4th** |
| --- | --- | --- | --- | --- | --- |
| **AR** | **Low structure** | 5 | 4 | 3 | 5 |
|  | **High structure** | 5 | 4 | 4 | 4 |
| **Non-AR** | **Low structure** | 5 | 5 | 4 | 3 |
|  | **High structure** | 2 | 4 | 6 | 5 |

**Enjoyment Additional Results**

**A**

**B**

**Supplementary Figure 1.** Number of child (A) and caregiver (B) responses on Enjoyment Smileyometer questionnaire following each condition.

**Supplementary Table 2.** Fun sorter number of responses for each condition.

|  | | **AR** | | **non-AR** | |
| --- | --- | --- | --- | --- | --- |
|  |  | **Low Structure** | **High Structure** | **Low Structure** | **High Structure** |
|  | | Number of responses | | | |
| **1^st^ most enjoyable** | **Child** | 15 | 11 | 8 | 0 |
|  | **Caregiver** | 21 | 6 | 5 | 2 |
| **2^nd^** | **Child** | 11 | 10 | 7 | 6 |
|  | **Caregiver** | 8 | 13 | 9 | 4 |
| **3^rd^** | **Child** | 7 | 7 | 11 | 9 |
|  | **Caregiver** | 3 | 13 | 9 | 9 |
| **4^th^ least enjoyable** | **Child** | 1 | 6 | 8 | 19 |
|  | **Caregiver** | 2 | 2 | 11 | 19 |

**Physical Activity Additional Results**

**Supplementary Figure 3.** Child’s perceived physical activity level number of responses for each condition.

**Supplementary Figure 4.** Accelerometer results for time (seconds) spent in low (sun of lie, sit, stand, and move), moderate (walking) and high (sum of running and stairs +) intensity physical activity in each condition.

**Supplementary Figure 5.** Accelerometer results for time (seconds) spent in different postures and movements in each condition.

**Accelerometer - Time (seconds) spent in postures and movements**

*Low Intensity*

There was a main effect of Animal Search (F(1, 33) = 110.24, p < 0.001) and Activity Structure (F(1,33) = 107.50, p < 0.001) on time spent in low intensity postures and movements. With longest spent during low structure AR compared to all other conditions (179 seconds, p < 0.001), followed by low structure non-AR (118 seconds) which differed compared to high structure non-AR (t(33) = 7.29, p < 0.001) but not high structure AR (t(33) = 0.68), p = 0.503)) (supplementary Figure 4). During low structure AR more time was spent in sitting and standing compared to all other conditions (p < 0.05) and moving compared to all except low structure non-AR. Sitting, standing and moving time were longer in high structure AR compared to high structured non-AR.

*Moderate Intensity*

There was no main effect of Animal Search (F(1, 33) = 0.54, p = 0.466) on time spent in moderate intensity movements but there was a main effect of Activity Structure (F(1, 33) = 67.65, p < 0.001) and an interaction effect (F(1, 33) = 12.24, p = 0.001). Time spent walking was longer in low structure compared to high structure conditions. Low structure AR and non-AR did not differ in walking time (t(33) = -1.78, p = 0.084) but high structure conditions did differ from each other with greater walking time in the high structure AR (t(33) = 4.01, p < 0.001).

*High Intensity*

There was a main effect of Animal Search (F(1, 33) = 16.1, p < 0.001), Activity Structure (F(1,33) = 16.2, p < 0.001) and an interaction effect (F(1, 33) = 11.7, p = 0.002) for time spent in high intensity movements. Longest was spent during low structure non-AR (91.2 seconds) compared to all other conditions (p < 0.001). Average time spent in high intensity movements did not differ between low structure AR, high structure AR and high structure non-AR (p > 0.05). The high intensity movement time in low structure non-AR was due to increased time spent running compared to all other conditions, as time spent in stairs+ did not differ. The least time spent running was during the high structure AR condition.

**Supplementary Table 3.** Accelerometer results for time in seconds spent in different postures and movements.

|  | **AR** | | | | **non-AR** | | | |
| --- | --- | --- | --- | --- | --- | --- | --- | --- |
|  | **Low Structure** | | **High Structure** | | **Low Structure** | | **High Structure** | |
| **Physical Activity** | | | | | | | | |
| **Low Int. (sec)** |  |  |  |  |  |  |  |  |
| **Lie** | 7.2 | ± 16.2^ac^ | 2.4 | ± 7.6^b^ | 8.9 | ± 17.5^ac^ | 2.7 | ± 8.6^ab^ |
| **Sit** | 37.1 | ± 26.6^a^ | 21.4 | ± 27.1^b^ | 12.4 | ± 14.7^b^ | 5.8 | ± 10.1^c^ |
| **Stand** | 38.7 | ± 26.7^a^ | 16.3 | ± 15.6^b^ | 10.9 | ± 13.2^b^ | 4.8 | ± 6.9^c^ |
| **Move** | 96.1 | ± 21.7^a^ | 71.4 | ± 32.2^b^ | 85.4 | ± 33.2^a^ | 43.3 | ± 26.7^c^ |
| **Total Low** | 179.1 | ± 40.4^a^ | 111.4 | ± 57.5^b^ | 117.7 | ± 46.6^b^ | 56.6 | ± 45.0^c^ |
| **Mod. Int.** |  |  |  |  |  |  |  |  |
| **Walk** | 90.0 | ± 29.9^a^ | 68.8 | ± 32.8^b^ | 103.3 | ± 41.0^a^ | 49.3 | ± 30.8^c^ |
| **Total Mod.** | 90.0 | ± 29.9^a^ | 68.8 | ± 32.8^b^ | 103.3 | ± 41.0^a^ | 49.3 | ± 30.8^c^ |
| **High Int.** |  |  |  |  |  |  |  |  |
| **Run** | 38.4 | ± 34.6^a^ | 23.7 | ± 18.3^b^ | 72.7 | ± 45.8^c^ | 38.2 | ± 23.9^a^ |
| **Stairs+** | 22.1 | ± 20.3^a^ | 24.23 | ± 15.3^a^ | 18.5 | ± 17.0^ab^ | 13.6 | ± 11.2^b^ |
| **Total High** | 60.6 | ± 36.2^a^ | 48.0 | ± 21.0^a^ | 91.2 | ± 49.6^b^ | 51.8 | ± 25.6^a^ |
|  |  |  |  |  |  |  |  |  |
